# Applications of rare earth elements in cancer: Evidence mapping and scientometric analysis

**DOI:** 10.3389/fmed.2022.946100

**Published:** 2022-08-10

**Authors:** Jinyu Wang, Sheng Li

**Affiliations:** ^1^School of Basic Medical Science, Lanzhou University, Lanzhou, China; ^2^The First People’s Hospital of Lanzhou City, Lanzhou, China

**Keywords:** rare earth elements, cancer, evidence mapping, scientometric analysis, VOS viewer

## Abstract

Cancer is one of the most important public health issues worldwide. Radiation therapy (XRT), chemotherapy, and targeted therapy are some of the main types of cancer therapy. Metals are used extensively in cancer diagnosis and therapy, and rare earth elements occupy an important niche in these areas. In recent years, an increasing number of studies have focused on the application of lanthanides in cancer diagnosis and therapy. However, no research has analyzed the current status and future trends of lanthanides in treating cancer. We downloaded data from publications from the Web of Science Core Collection. We used VOSviewer 1.16.16 software and Excel 2016 to analyze literature information, including publication years, journals, countries, institutes, authors, keywords, and co-cited references. A total of 7,849 publications were identified. The first study on the association of rare earth elements with cancer was published in 1945. However, before 1979, the number of publications per year was no more than 10. After 1980, the number of yearly publications increased. The United States was the most productive country (2,726, 34.73%), and the institution with the most frequent contributions was the Chinese Academy of Sciences (211, 2.69%). We observed close collaboration between countries and between institutes. The 7,839 publications were published in 1,579 journals, and Radiology was both the most productive journal (183, 2.33%) and cited journal (5,863 citations). A total of 33,987 authors investigated rare earth elements and cancer. Only 0.45% of the authors published more than 10 publications, and 79.07% of the authors published only one publication. Of the top 10 high-yield authors, seven were from developed countries and three were from China. However, among the top 10 co-cited authors, there was only one high-yield author. The main research topics in the application of lanthanide complex-doped nanomaterials in the diagnosis and treatment of cancer include magnetic resonance imaging contrast agents, photodynamic therapy, anticancer drug delivery, the efficacy and safety of yttrium-90 radioimmunotherapy and chemoembolization for the treatment of HCC, gadolinium magnetic resonance imaging (MRI) contrast agent for cancer diagnosis, and cerium oxide nanoparticles. In recent years, especially since 2016, the research frontiers are emerging in cerium oxide nanoparticles and photodynamic therapy. Studies related to the application of rare earth elements and cancer have significantly increased over the past 20 years. The United States contributed the most articles in the field, followed by China and Germany, and cooperation among countries was frequent. The Chinese Academy of Sciencess, Northwestern University, and Stanford University were the three most productive institutions, and cooperation among institutions was frequent. Many high-quality journals have published relevant research, but there are few highly productive journals.

## Highlights

Question: What are the current status and future trends of lanthanides in treating cancer?

Pertinent findings: In this evidence mapping and scientometric analysis, we found that studies related to the application of rare earth elements and cancer have significantly increased over the past 20 years. The United States contributed the most articles in the field, followed by China and Germany, and cooperation among countries was frequent. The Chinese Academy of Sciencess, Northwestern University, and Stanford University were the three most productive institutions, and cooperation among institutions was frequent. Many high-quality journals have published relevant research, but there are few highly productive journals.

Implications for patient care: The application of lanthanide complex-doped nanomaterials in the diagnosis and treatment of cancer includes magnetic resonance imaging contrast agents, photodynamic therapy, anticancer drug delivery, the efficacy and safety of yttrium-90 radioimmunotherapy and chemoembolization for the treatment of HCC, gadolinium magnetic resonance imaging (MRI) contrast agents for the diagnosis of cancer, and cerium oxide nanoparticles are the main research topics in this field.

## Introduction

Cancer is one of the most important public health issues worldwide (1) million cancer deaths in 2020 worldwide (2). It is reported that the oldest evidence of cancer was found in the remains of a 4,200-year-old Egyptian woman (3). Because cancer has a long history, there have been many different medical diagnostic and therapeutic approaches and many biological and clinical advances have been made (4). Radiation therapy (XRT), chemotherapy, and targeted therapy are some of the main types of cancer therapies (5). Metals are used extensively in cancer diagnosis and therapy, and rare earth elements occupy an important niche in these areas (5).

Rare earth elements are also known as lanthanides, which indicate elements with atomic numbers ranging from 57 to 71 and have been of interest since the 19th century (5, 6). Because the biological properties of rare earth element ions are similar to those of calcium ions, rare earth elements are used in medicine research (6). Cerium is one of the major rare earth elements, and cerium oxalate was the earliest therapeutic application of rare earth elements in medicine as an antiemetic agent (6). The preparation of active rare earth ion complexes was first described in the mid-19th century and used until the middle of the 1900s (6). From the beginning of the 20th century, rare earth elements have been used in different fields of medicine; e.g., salts of rare earth metal ions were used for the treatment of tuberculosis, lanthanum chloride was used for its anti-atherosclerotic effects, and lanthanum carbonate for the treatment of hyperphosphatemia (7–9).

Lanthanide coordination compounds play an important role in cancer diagnosis and therapy, and they can also be used as antibacterial agents (6). Gadolinium complexes, including gadopentetic acid and gadoteric acid, are widely used in imaging diagnostics, especially in magnetic resonance imaging (MRI) contrast agents for cancer imaging (10). Lanthanide radioisotopes (e.g., 177 Lu) have been used in cancer imaging and therapy (11). Other lanthanides such as Nd, Sm, and Yb, are good factors for *in vivo* luminescence imaging (12). Moreover, lanthanide oxide nanoparticles, nanodrums, and nanocrystals are considered promising potential imaging and anti-cancer agents (13).

In recent years, an increasing number of studies have focused on the applications of rare earth elements in cancer diagnosis and therapy (5, 6, 13–15). However, no research has analyzed the current status and future trends of rare earth elements in cancer. Our study aimed to analyze the distribution of years, authors, countries, institutions, and journals on the research of rare earth elements in cancer, identify the cooperation between countries and between institutions, and explore existing topics of interest and future prospects.

## Materials and methods

### Source of literature

We systematically searched the Science Citation Index-Expanded of the Web of Science (WoS) Core Collection from inception to November 4, 2021. The detailed search strategy was as follows: TS = “rare earth element” OR lanthanide OR cerium OR scandium OR Yttrium OR Lutetium OR Ytterbium OR Thulium OR Erbium OR Holmium OR Dysprosium OR Terbium OR Gadolinium OR europium OR Samarium OR Promethium OR Neodymium OR Praseodymium, and TS = cancer OR tumor OR carcinoma OR neoplasm. In our study, we considered any type of study, and there were no restrictions on publication year, language, or data category. To avoid the bias caused by the daily database updates, we conducted all searches on the same day. We exported records in CSV format from the database and all records would contain title, language, journal, author, year of publication, affiliation, keywords, document type, abstract, and counts of citation.

### Statistical analysis

VOSviewer software version 1.6.16 was used to generate social network maps and perform cluster analysis, which analyzed frequently used keywords, top productive research institutions, authors, and countries, co-cited authors, co-cited journals, and co-cited references. Network maps consist of many links and nodes. Co-cited authors are authors that have been cited together, co-cited references are references means that references are co-cited in a set of publications. Different nodes represent different elements, including keywords, institutions, and countries. The larger the size of the nodes, the greater the frequency or number of publications. Different nodes were connected with different links, which represented relationships of collaboration, co-occurrence, or co-citations. The colors of the nodes and lines represented different clusters or years. Microsoft Excel 2019 was used to analyze the publication trends. A trinomial polynomial model was applied to forecast the growth of publications in the following year. We have standardized and adjusted some words during the analysis, e.g., we reclassified articles from England, Northern Ireland, Scotland, and Wales to the United Kingdom and articles from Hong Kong, Macau, and Taiwan to China.

### Parameter setting

The parameters of VOSviewer were the counting method (fractional counting) and data type (bibliographic data). We ignored documents with a large number of authors; the maximum number of authors per document was 25.

## Results

### Language and document types of publication

A total of 7,849 publications were identified in the Science Citation Index-Expanded of the WoS, which were published in 15 languages. More than 7,600 publications (97.97%) were published in English, followed by unspecified (95, 1.21%), German (83, 1.06%), French (46, 0.59%), Chinese (18, 0.23%), Russian (13, 0.17%), Spanish (11, 0.14%), Korean (7, 0.09%), Italian (4, 0.05%), Japanese (4, 0.05%), Portuguese (2, 0.03%), Turkish (2, 0.03%), Cezch (1, 0.01%), Dutch (1, 0.01%), and Hungarian (1, 0.01%). The 7,849 included studies were divided into 19 document types. Articles were the main document type, accounting for 80.83% of the included studies. The remaining document types were other (2547, 32.45%), meeting abstracts (2560, 17.23%), reviews (543, 6.92%), case reports (400, 5.10%), clinical trials (349, 4.45%), editorial materials (127, 1.62%), letters (116, 1.48%), unspecified (50, 0.64%), early access (39, 0.50%), corrections (20, 0.26%), books (9, 0.12%), news (6, 0.08%), data sets (3, 0.04), reference materials (2, 0.03%), retracted publications (3, 0.03%), and reports (1, 0.01%).

### Annual publications and growth forecast

**FIGURE 1:**
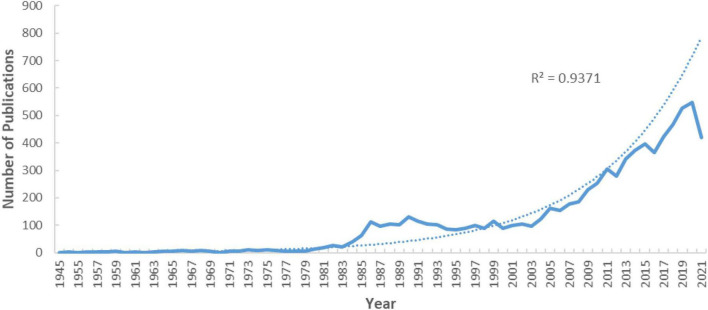
Publication years and growth forecast.

The first study that included applications of rare earth elements to cancer diagnosis and treatment was published in 1945 (Figure 1). However, before 1979, the number of publications per year was no more than 10. After 1980, the number of publications increased and reached 100 publications in 1986, 229 publications in 2009, 343 publications in 2013, 423 publications in 2017, and 528 publications in 2019. From 2009 to 2019, 4,930 studies on the association of rare earth elements with cancer were published, accounting for 62.81% of the included studies.

We also used the three-term polynomial model to assess the relationship between the growth of publications and publication year (Figure 1). We observed a significant correlation between the year and the number of publications with a high coefficient of determination (R^2^ = 0.9371). From the fitting curve, we can predict that the annual articles will continue to grow in the coming years.

### Countries and institutions

**FIGURE 2:**
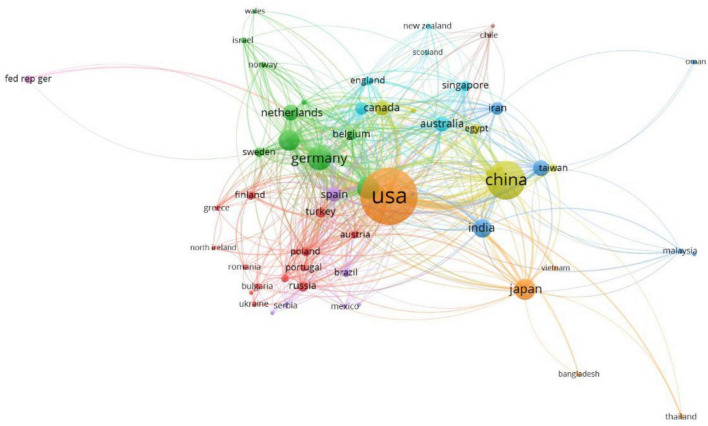
The network map of countries.

In total, 101 countries were involved in research on the association of rare earth elements with cancer. The United States published the most publications, accounting for 34.73%, followed by China (1246, 16.10%), Germany (509, 6.48%), Italy (391, 4.98%), France (367, 4.76%), Japan (366, 4.66%), India (282, 3.59%), South Korea (228, 2.90%), Netherlands (209, 2.66%), and Canada (207, 2.64%) (Table 1). Figure 2 presents the analysis of the social relations of countries with more than five publications. Sixty countries that published more than five articles could be clustered into nine categories showing active cooperation.

**TABLE 1:** The top 10 authors and co-cited authors in the research on the association of rare earth elements with cancer [n (%)].

Rank	Authors	N (%)	Co-cited authors	Citations
1	Salem R	83 (1.06)	Salem R	820 (10.45)
2	Lewandowski R. J	52 (0.66)	Wang F	552 (7.03)
3	Seal S	35 (0.45)	Caravan P	417 (5.31)
4	Tillement O	30 (0.38)	Aime S	354 (4.47)
5	Chen XY	28 (0.36)	Bunzli JCG	351 (4.45)
6	Miller R	28 (0.36)	Chen GY	340 (4.33)
7	Semelka R	28 (0.36)	Kennedy AS	316 (4.03)
8	Lux F	27 (0.34)	Zhou J	308 (3.92)
9	Lam M G. E. H	26 (0.33)	Runge VM	294 (3.75)
10	Lin J	26 (0.33)	Kostova I	283 (3.61)

**FIGURE 3:**
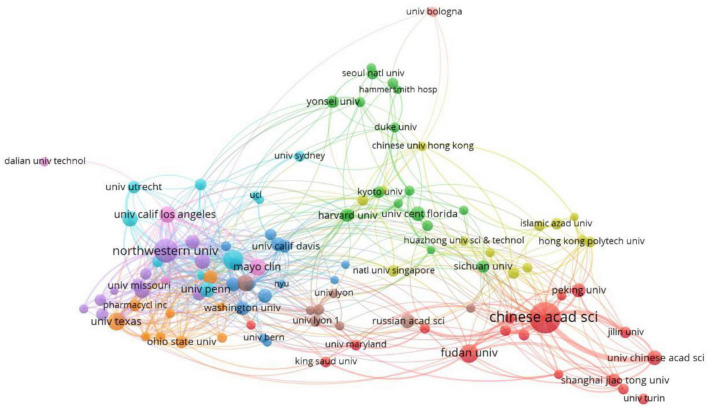
The network map of institutions.

In general, 5,548 institutions contributed to research on the association of rare earth elements with cancer. Table 2 shows the top 10 institutions, and the Chinese Academy of Sciences ranked first (211, 2.69%), followed by Northwestern University (128, 1.63%), Stanford University (89, 1.13%), Fudan University (77, 0.98%), University of Texas (76, 0.97%), Mayo Clinic (71, 0.90%), National Cancer Institute (63, 0.80%), University of California Los Angeles (62, 0.79%), University of Pittsburgh (59, 0.75%), and the University of Pennsylvania (58, 0.74%). The collaborations of the institutions are presented in Figure 3. From the Figure, we can see that the cooperation among the main institutions was close.

**TABLE 2:** The top 10 countries and institutions contributed to publications in the research on the association of rare earth elements with cancer [n (%)].

Rank	Country	N (%)	Institution	N (%)
1	United States	2,726 (34.73)	Chinese Academy of Sciences (China, Beijing)	211 (2.69)
2	China	1,246 (16.10)	Northwestern University (United States, Evanston)	128 (1.63)
3	Germany	509 (6.48)	Stanford University (United States, Palo Alto)	89 (1.13)
4	Italy	391 (4.98)	Fudan University (China, Shanghai)	77 (0.98)
5	France	367 (4.76)	University of Texas (United States, Texas)	76 (0.97)
6	Japan	366 (4.66)	Mayo Clinic (United States, Rochester)	71 (0.90)
7	India	282 (3.59)	National cancer institute (United States)	63 (0.80)
8	South Korea	228 (2.90)	University of California. Los Angeles (United States, Los Angeles)	62 (0.79)
9	Netherland	209 (2.66)	University of Pittsburgh (United States, Pittsburgh)	59 (0.75)
10	Canada	207 (2.64)	University of Pennsylvania (United States, Philadelphia)	58 (0.74)

### Authors and co-cited authors

**FIGURE 4:**
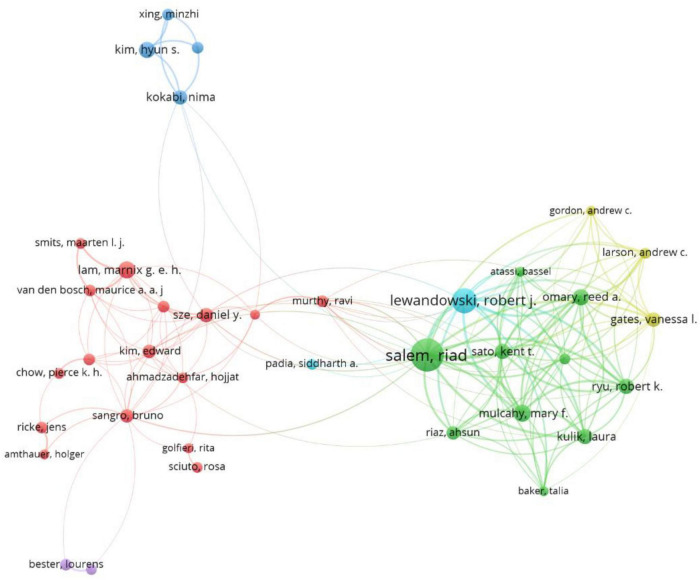
The network map of authors.

A total of 33,987 authors participated in the research on the association of rare earth elements with cancer, and the top 10 authors and co-cited authors are presented in Table 1. Salem R ranked first with 83 publications (1.06%), followed by Lewandowski (52, 0.66%), Seal S (35, 0.45%), and Tillement O (30, 0.38%). The remaining authors published fewer than 30 studies. The top three co-cited authors were Salem R (820, 10.45%), Wang F (552, 7.03%), and Caravan P (417, 5.31%). Other authors were cited fewer than 400 times. Figure 4 shows the collaborations of authors with more than 10 articles.

### Journals and co-cited journals

A total of 7,849 identified publications were published in 1,579 journals, and the top 10 journals and co-cited journals are presented in Table 3. *Radiology* with 183 studies published the most articles, followed by *the Journal of Nuclear Medicine* (173, 2.20%), and the *Journal of Vascular and Interventional Radiology* (139, 1.77%). Seven of the top 10 journals were from the United States, and the other three journals were from Netherlands and Germany, respectively. The impact factors of the four journals were greater than 10. *Radiology* was also the most co-cited journal with 5,863 citations, followed by the *Journal of the American Chemical Society* (5,611 citations), and *Biomaterials* (4,127 citations). Of the top 10 co-cited journals, seven were from the United States and one from the United Kingdom, Netherlands, and Germany.

**TABLE 3:** The top 10 journals and co-cited journals in the research on the association of rare earth elements with cancer [n (%)].

Rank	Journals	N (%)	Country	IF (2020)	Co-cited journals	Co-citation	Country	IF (2020)
1	Radiology	183 (2.33)	United States	11.105	Radiology	5,863	United States	11.105
2	Journal of nuclear medicine	173 (2.20)	United States	10.057	Journal of the American Chemical Society	5,611	United States	15.419
3	Journal of vascular and interventional radiology	139 (1.77)	United States	3.464	Biomaterials	4,127	Netherlands	12.479
4	The journal of urology	139 (1.77)	Netherlands	7.45	ACS Nano	3,563	United States	15.881
5	Blood	132 (1.68)	United States	22.113	Angewandte chemie-international edition	3,545	Germany	15.336
6	International journal of radiation oncology biology physics	122 (1.55)	Netherlands	7.038	Journal of nuclear medicine	3,526	United States	10.057
7	European journal of nuclear medicine and molecular imagine	103 (1.31)	Germany	9.236	Advanced materials	2,404	United States	30.849
8	Journal of clinical oncology	102 (1.30)	United States	44.544	Chemical reviews	2,356	United States	60.622
9	Lasers in surgery and medicine	100 (1.27)	United States	4.025	Chemical society review	2,317	United Kingdom	54.564
10	Journal of endourology	78 (1.00)	United States	2.942	Journal of clinical oncology	2,272	United States	44.544

### Co-occurrence keywords

**FIGURE 5:**
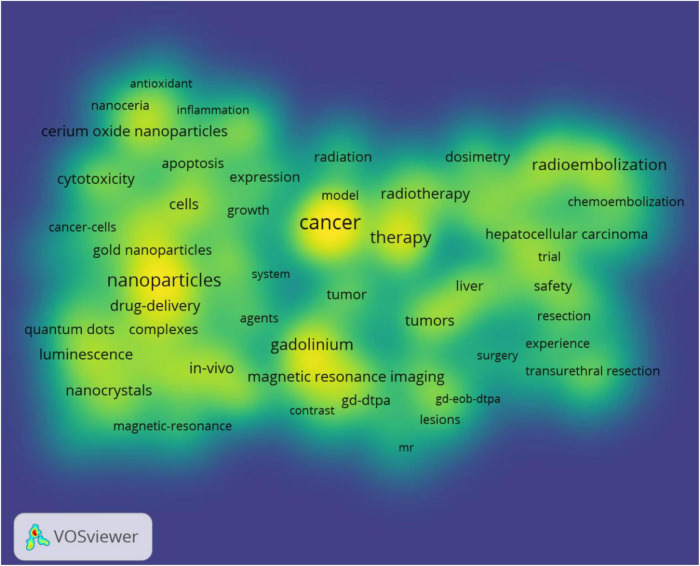
The density map of keywords.

Keywords with a higher frequency could accurately indicate the main topic of a field. In our study, Gadolinium is the most often rare earth element, followed by Yttrium, Lanthanum, Cerium, and Holmium. Additionally, Table 4 presents the top 10 rare earth elements in the research on the association of rare earth elements with cancer. A total of 17,770 keywords appeared, but only 146 keywords had over 150 frequencies. Figure 5 and Table 5 present the results of the analysis according to co-occurrence frequency, cancer, nanoparticles, therapy, gadolinium, Magnetic Resonance Imaging, radioembolization, cells, contrast agents, *in vivo*, luminescence, and cerium oxide nanoparticles were the most common keywords.

**TABLE 4:** The top 10 rare earth elements in the research on the association of rare earth elements with cancer.

Rank	Rare earth elements	*N*
1	Gadolinium	492
2	Lanthanum	385
3	Yttrium	353
4	Cerium	261
5	Holmium	121
6	Lutetium	107
7	Europium	89
8	Neodymium	48
9	Thulium	46
10	Ytterbium	35

**TABLE 5:** The top 20 keywords in terms of frequency in the research on the association of rare earth elements with cancer.

Rank	Keywords	*N*	Rank	Keywords	*N*
1	Cancer	948	11	Cerium Oxide Nanoparticles	231
2	Nanoparticles	502	12	Toxicity	225
3	Therapy	467	13	Photodynamic therapy	222
4	Gadolinium	341	14	*In vitro*	221
5	Magnetic Resonance Imaging	566	15	Survival	218
6	Radioembolization	281	16	Oxidative stress	217
7	Cells	257	17	Hepatocellular-carcinoma	210
8	Contrast agents	254	18	Radiotherapy	209
9	*In vivo*	244	19	Drug-delivery	202
10	Luminescence	240	20	Chemotherapy	201

**FIGURE 6:**
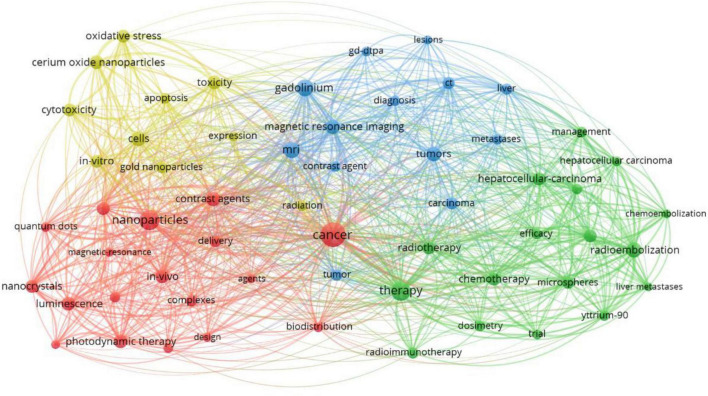
The network map of keywords.

**FIGURE 7:**
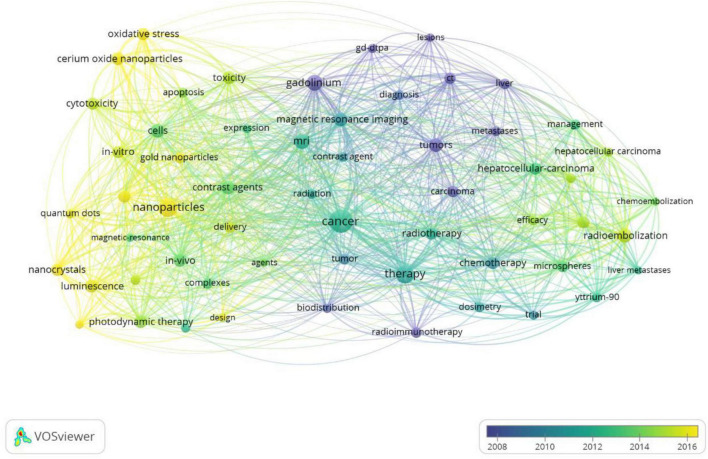
The overlay map of keywords.

Figure 6 shows the clustering analysis of co-occurrence keywords with frequencies greater than 100. In addition, 58 keywords were divided into four categories. Cluster 1 included 18 items and mainly focused on lanthanide complexes, nanoparticles, contrast agents, nanocrystals, and nanocrystals. Cluster 2 included 17 items and mainly focused on chemotherapy, hepatocellular carcinoma, radioimmunotherapy, yttrium-90, safety, and efficacy. Cluster 3 included 13 items and mainly focused on contrast agents, gadolinium, and magnetic resonance imaging. Cluster 4 included 10 items and mainly focused on cerium oxide nanoparticles, cytotoxicity, radiation, and gold nanoparticles. Figure 7 presents the overlay map of keywords with more than 100 frequencies, which showed that from 2016, the research frontiers mainly focused on oxidative stress, cerium oxide nanoparticles, quantum dots, drug delivery, nanoparticles, luminescence, and photodynamic therapy. Additionally, Figure 7 also showed that although Gadolinium is the most often element, it is not the element of research frontiers. After 2016, Cerium became a research frontier.

### Co-cited references

Table 6 presents the top 10 co-cited references in terms of research on the association of rare earth elements with cancer. One study was co-cited more than 200 times, two articles were co-cited between 120 and 140 times, five studies were co-cited 114–119 times, and two articles were co-cited 104 times.

**TABLE 6:** The top 10 co-cited references in terms of the research on the association of rare earth elements with cancer [n (%)].

Rank	Co-cited references	Co-citation
1	Caravan P, 1999. Gadolinium (III) Chelates as MRI Contrast Agents: Structure, Dynamics, and Applications. Chem Rev. V99, P2293.	239
2	Salem R, 2006. Radioembolization with 90 Yttrium microspheres: a state-of-the-art brachytherapy treatment for primary and secondary liver malignancies. Part 1: Technical and methodologic considerations. J Vasc Interv Radiol. V17, P1251.	140
3	Pirmohamed T, 2010. Nanoceria exhibit redox state-dependent catalase mimetic activity. Chem Commun (Camb). V4, P2736.	120
4	Weinmann HJ, 1984. Characteristics of gadolinium-DTPA complex: a potential NMR contrast agent. AJR Am J Roentgenol. V142, P619	119
5	Korsvik C, 2007. Superoxide dismutase mimetic properties exhibited by vacancy engineered ceria nanoparticles. Chem Commun (Camb). V142, P619	117
6	Mosmann T, 1983. Rapid colorimetric assay for cellular growth and survival: application to proliferation and cytotoxicity assays. J Immunol Methods. V65, P55	117
7	Kennedy A, 2007. Recommendations for radioembolization of hepatic malignancies using yttrium-90 microsphere brachytherapy: a consensus panel report from the radioembolization brachytherapy oncology consortium. Int J Radiat Oncol Biol Phys. V68, P13	116
8	Tarnuzzer RW, 2005. Vacancy engineered ceria nanostructures for protection from radiation-induced cellular damage. Nano Lett. V5, P2573	114
9	Salem R, 2010. Radioembolization for hepatocellular carcinoma using Yttrium-90 microspheres: a comprehensive report of long-term outcomes. Gastroenterology. V138, P52	104
10	Bünzli JC, 2010. Lanthanide luminescence for biomedical analyses and imaging. Chem Rev. V110, P2729	104

## Discussion

We systematically searched the Web of Science and identified 7,849 articles. The first relevant research was published in 1945; however, in the 30 years after, there were only 31 articles related to the application of rare earth elements in cancer, which indicates that the application of rare earth elements in cancer developed slowly during this period. After 1980, the number of publications increased; the possible reasons include that since the beginning of the 20th century, rare earth elements were used in medicine (6–9). Over 77.96% of the articles were published between 2000 and 2021. With the continuous improvement of methods for the extraction and separation of lanthanide salts, an increasing number of researchers, countries, and scholars have focused on utilizing rare earth elements in cancer imaging and treatment, which has led to more abundant relevant research in recent years. A significant positive relationship was found between the year and the number of publications, and it is expected that research on the application of rare earth elements in cancer will continue to grow in the coming years.

A total of 33,987 authors investigated rare earth elements and cancer. Only 0.45% of the authors published more than 10 publications, and 79.07% of the authors published only one publication, which indicates that although an increasing number of authors participated in research on the application of rare earth elements in cancer, there are few high-yield authors. Of the top 10 high-yield authors, seven were from developed countries and three were from China. However, among the top 10 co-cited authors, there was only one high-yield author. Relevant authors should strengthen cooperation to innovate research methods and improve the quality of the studies.

Among the top 10 countries contributing to relevant publications, only one was a developing country, which indicates that research on the application of rare earth elements in cancer is relatively insufficient and backward. Therefore, developing countries need to cooperate with developed countries to learn advanced technology methods and research to promote the development of relevant research worldwide. Moreover, available funding for conducting relevant research is also needed for researchers in developing countries to increase the research output on rare earth elements and cancer. Among the top 10 institutions, eight were from the United States, and most were American universities. The remaining two were from China.

A total of 7,849 articles were published in 1,579 journals, but only 9.69% of journals published more than 10 papers, and 51.60% of journals only published one relevant paper, which reveals that many journals contributed to the publication of the application of rare earth elements and cancer, but journals insisted that publication of relevant articles was insufficient. Of the top 10 journals, six were from the United States and the mean impact factors of the six journals were greater than 16.41, and the impact factors of the four from the United States were greater than 10. Journals from the United States publish studies of rare earth elements and cancer most frequently, and the impact factors of these high-yield journals are also high. Among the top 10 co-cited journals, only three were also highly productive journals; *Radiology was* the journal with the highest number of relevant articles and was also the most co-cited journal. The impact factors of all of the top 10 co-cited journals were greater than 10, indicating that studies published in high-impact factor journals were cited more often. A possible reason for this phenomenon might be that higher-impact journals have higher requirements for topic selection and quality of published articles.

In bibliometrics, a network graph of keyword co-occurrences can reflect topics of interest (16). In this study, 17,770 keywords were generated, and 146 keywords were appearing over 50 times, revealing that a higher frequency of a fewer number of keywords could better indicate the main topic of interest in a field. We used the VOS viewer to perform cluster analysis for co-occurrence keywords and four topics were generated: cluster 1 contained 18 keywords, which mainly focused on lanthanide complex-doped nanomaterials applied in the diagnosis and treatment of cancer, including magnetic resonance imaging contrast agents, photodynamic therapy, and anticancer drug delivery. Nanomaterials with unique surfaces and internal structures showed improved circulation and reduced toxicity (17). Up-conversion nanoparticles can convert near-infrared (NIR) light at long wavelengths into visible (Vis) and even UV light with shorter wavelengths (18), and thus have many advantages including minimal photodamage, deep tissue penetration, absence of autofluorescence, weak photobleaching, and good chemical stability (19–21). Nanomaterials have been confirmed to have excellent application potential in cancer therapy (17). Cluster 2 included 17 keywords, mainly related to the efficacy and safety of yttrium-90 radioimmunotherapy and chemoembolization for the treatment of hepatocellular carcinoma (HCC). HCC is one of the most common and deadly cancers worldwide (22, 23). More than 70% of patients with HCC cannot undergo curative surgery; locoregional therapies, including chemoembolization, radioembolization, and radioimmunotherapy, are generally used to control the progress of the disease in patients with HCC globally (24–27). Previous studies showed that Yttrium-90 radioembolization and radioimmunotherapy are associated with better clinical outcomes in patients with HCC (28, 29). Cluster 3 contained 13 keywords focused on gadolinium magnetic resonance imaging (MRI) contrast agents for the diagnosis of cancer. Lanthanide plays an important role in the field of medicine, especially in cancer diagnosis and treatment. In recent years, gadolinium complexes have been widely used in MRI for diagnostic purposes and monitoring treatment progress (30–33). Cluster 4 included 10 keywords mainly concerned with cerium oxide nanoparticles in the role of enhancing radiation-induced and chemotherapy-induced cancer apoptosis. When conventional treatments including surgery, radiation, and cytotoxic chemotherapies are ineffective in cancer treatment, nanomedicines are one of the most recently developed treatment options (33–37). Cerium oxide nanoparticles consist of a cerium core surrounded by an oxygen lattice, which plays an important role in antioxidant behavior. Previous studies have indicated that cerium oxide nanoparticles have extensive potential as a therapeutic agent for the treatment of cancer because cerium oxide nanoparticles possess innate cytotoxicity to cancer cells, anti-invasive properties, and the ability to sensitize cancer cells to radiation-induced cell death while protecting the surrounding normal tissues (38–41).

### Strengths and limitations

To the best of our knowledge, this study is the first bibliometric analysis of the application of rare earth elements for cancer. We clearly show the current status and future trends of relevant research by conducting frequency analysis, cluster analysis, and hotspot analysis.

There are some limitations to our study. First, because WOS is the most important source of data for bibliometric analysis in science (42), we only searched WOS in this study, which could result in insufficient and unrepresentative studies that were included in the current study. Second, most of the identified studies were published in English, which may have led to language bias. Third, since there are many authors and keywords in this study, although we have standardized and adjusted during the analysis, the omission may still exist.

## Conclusion

Studies related to the application of rare earth elements and cancer have significantly increased over the past 20 years. The United States contributed the most articles in the field, following China and Germany, and cooperation among countries was close. *The Chinese Academy of Sciencess, Northwestern University*, and *Stanford University* were the top three productive institutions, and cooperation among institutions was active. Many high-quality journals have published relevant research, but there are few highly productive journals. The application of lanthanide complex-doped nanomaterials in the diagnosis and treatment of cancer includes magnetic resonance imaging contrast agents, photodynamic therapy, anticancer drug delivery, the efficacy and safety of yttrium-90 radioimmunotherapy and chemoembolization for the treatment of HCC, gadolinium magnetic resonance imaging (MRI) contrast agents for the diagnosis of cancer, and cerium oxide nanoparticles are the main research topics in this field. In recent years, especially since 2016, research frontiers include cerium oxide nanoparticles and photodynamic therapies.

## Data availability statement

The original contributions presented in this study are included in the article/supplementary material, further inquiries can be directed to the corresponding author/s.

## Author contributions

SL and JW conceived and designed the study, screened and selected the articles, interpreted the data, and drafted the manuscript. Both authors had full access to all the data in the study and had final responsibility for the decision to submit for publication.
